# *De novo* assembly of highly diverse viral populations

**DOI:** 10.1186/1471-2164-13-475

**Published:** 2012-09-13

**Authors:** Xiao Yang, Patrick Charlebois, Sante Gnerre, Matthew G Coole, Niall J Lennon, Joshua Z Levin, James Qu, Elizabeth M Ryan, Michael C Zody, Matthew R Henn

**Affiliations:** 1 The Broad Institute of MIT and Harvard, Cambridge, MA 02142 USA

## Abstract

**Background:**

Extensive genetic diversity in viral populations within infected hosts and the divergence of variants from existing reference genomes impede the analysis of deep viral sequencing data. A *de novo* population consensus assembly is valuable both as a single linear representation of the population and as a backbone on which intra-host variants can be accurately mapped. The availability of consensus assemblies and robustly mapped variants are crucial to the genetic study of viral disease progression, transmission dynamics, and viral evolution. Existing *de novo* assembly techniques fail to robustly assemble ultra-deep sequence data from genetically heterogeneous populations such as viruses into full-length genomes due to the presence of extensive genetic variability, contaminants, and variable sequence coverage.

**Results:**

We present *VICUNA*, a *de novo* assembly algorithm suitable for generating consensus assemblies from genetically heterogeneous populations. We demonstrate its effectiveness on Dengue, Human Immunodeficiency and West Nile viral populations, representing a range of intra-host diversity. Compared to state-of-the-art assemblers designed for haploid or diploid systems, *VICUNA* recovers full-length consensus and captures insertion/deletion polymorphisms in diverse samples. Final assemblies maintain a high base calling accuracy. *VICUNA* program is publicly available at: http://www.broadinstitute.org/scientific-community/science/projects/viral-genomics/ viral-genomics-analysis-software.

**Conclusions:**

We developed *VICUNA*, a publicly available software tool, that enables consensus assembly of ultra-deep sequence derived from diverse viral populations. While *VICUNA* was developed for the analysis of viral populations, its application to other heterogeneous sequence data sets such as metagenomic or tumor cell population samples may prove beneficial in these fields of research.

## Background

Viral borne diseases exert a significant impact on human health with millions of individuals infected yearly and diseases such as HIV/AIDS ranking as a leading cause of death worldwide ( http://www.who.int/mediacentre/factsheets/fs310/en/index.html). Critical to the design of effective vaccines and therapeutics to combat this burden is a comprehensive map of the genetic composition of viral populations and the characterization of the selective pressures that shape these populations. Compiling such a map is a challenge: in viral infections the low fidelity of the genome replication process and various evolutionary pressures can result in a single infected host harboring a heterogeneous population of genetic variants [1]. Previous studies [2-9] have shown that many viruses including Dengue, HCV, HIV, Influenza, Polio and West Nile all maintain diverse populations within a single host. This genetic diversity means that the population may already contain variants that are advantageous in the face of challenges such as host immune responses and drug treatments. As such, understanding the extent and composition of intra-host population diversity, even at low frequencies, can be very important in evaluating disease progression, transmission, and response to changes in therapy.

However, identifying intra-host variation depends on an alignment of the sequence data. In cases where the reads cover identical sequences, multiple alignment can be used to solve this problem [10], but this is currently not feasible for whole genome sequences as no platform exists to sequence whole genomes as single reads at high throughput. Alternatively, the sequencing data can be aligned to a reference, as is commonly done for variant detection in human and other organisms [11,12]. However, the use of a reference genome that is too genetically distant from the sample population will yield inaccurate read alignments and, as previously reported, substantial data loss; both factors decrease the ability to detect biological variants [13,14]. Because viral consensus can vary substantially between patients it may be difficult to find an existing reference that allows unbiased and complete alignment of reads [2,15]. One solution to this problem is to start the analysis by *de novo* assembly of each patient sample, allowing use of the patient consensus as the reference for variant detection [2]. Since the sample consensus will be near the centroid of the intra-host variation, it should be optimal for alignment of all sequence data to a single reference.

Further, the consensus sequence itself is of value. Notably, the majority of publicly available genomic sequences were captured using bulk Sanger sequencing strategies and as such a single consensus assembly is the only data available to compare against to derive biological insights. A consensus serves as a single datum that represents the entire underlying population or some subset of the population and thereby enables the identification of dominant genetic mutations that vary between two populations or subsets of the same population [2,15,16]. Lastly, for samples infected with unknown viruses a reference guided mapping strategy will not be applicable and a *de novo* approach is required [2,17,18].

There are two major frameworks for *de novo* genome assembly. Overlap-layout-consensus based methods [19-21] first identify reads that share good suffix-prefix alignment. This operation divides the input reads into disjoint sets, termed *contigs*. Then, multiple sequence alignment is performed for each contig to derive the consensus sequence, an approximation to a target genome fragment. The relative positions and orientations of these contigs are estimated by paired reads that land in different contigs. The de Bruijn graph based methods [13,22-27] first decompose input reads into *k*mers, denoted as vertices, then create a directed edge between any pair of vertices if the last (*k*−1) bases of the source vertex is identical to the first (*k*−1) bases of the target vertex. The graph is then simplified by chain compaction to shorten paths that have a unique entry and exit, and edited to remove small tips and bubbles that are likely attributed to sequencing errors. Finally, contigs are generated and oriented during graph traversal, guided by paired reads and coverage information.

Assembly algorithms devised specifically for the sequencing of less diverse haploid and diploid genomes by short reads tend to fare poorly on data derived from variant populations such as viruses, both because of the difficulty of separating continuous variation from error, and because of other process-related challenges [2]. In addition, sample preps of RNA viral genomes currently require reverse transcription and usually require amplification. These protocols tend to result in highly variable coverage along the length of the genome and may introduce large amounts of contamination from other RNA species present in the starting material. These artifacts of the process tend to further confuse existing assembly algorithms, which rely on depth of coverage to both indicate copy number of sequences and distinguish errors from true variants.

Recent work utilizing an overlap-layout-consensus strategy has shown that it is possible to generate high quality *de novo* assemblies from relatively deep coverage data produced by massively parallel pyrosequencing of diverse populations [2]. However, this method adapts poorly to other platforms such as the Illumina and Ion Torrent, due to a much larger amount of data produced, which increases both the computational complexity, and the difficulty of merging divergent genotypes and handling process error. An alternative strategy was recently reported by Iqbal *et al.*[13] These authors demonstrated *de novo* assembly of diploid genomes or a population consisting of a small number of eukaryotic genomes using a de Bruijn graph strategy, but the amount of diversity inherent in viral populations is beyond the target range for this algorithm.

Here we present *VICUNA*, a *de novo* assembler of very high but variable coverage short read data from a population of diverse but non-repetitive genomes. *VICUNA* is an overlap-layout-consensus based assembly algorithm. Unlike assemblers optimized for large repetitive genomes, *VICUNA* aggressively merges similar sequences, and has the capacity to retain low frequency single nucleotide and length polymorphisms. We validated *VICUNA* on 12 viral population samples. These 12 samples were obtained from patients infected with Dengue Virus (DENV), Human Immunodeficiency Virus (HIV) and West Nile Virus (WNV), which represent a spectrum of intra-host population variation. *VICUNA* recovers the full target regions with high fidelity in all samples. The algorithm captures low frequency non-dominant length variants, specified by a tunable threshold. In a handful of these samples, we recovered alternate consensus containing large deletions (≥ 500*bp*). *VICUNA* runs on workstations or blades, and hence is readily accessible to research labs with limited compute resources. Although our immediate target application is the sequencing of RNA viruses from infected hosts, we anticipate that our method may also have utility for a range of applications which pose similar challenges, including rRNA sequencing and whole genome metagenomic analyses.

## Results and discussion

### *VICUNA* assembly strategy

We assume a typical viral population consists of an unknown number of genomic variants that vary in their frequencies. A subset of these variants dominates the population and minor variants also exist. We assume that the phylogenetic relatedness within variant clusters is greater than that between clusters. Notably, the premise of a heterogeneous population comprised of closely and distantly related groups of sequences occurring in varied frequencies is applicable to other biological investigations such as metagenomics. The goal of generating a set of consensus sequences that represents a viral population is confounded by high genomic mutation rates, which results in frequent single nucleotide and length polymorphisms between genomes. This paralyzes the key error correction component of de Bruijn graph based assemblers. *VICUNA* applies the basic rule that a contig is produced to represent a spectrum of genomic fragments such that their pairwise genetic distances, defined as one hundred minus the percentage of sequence alignment identity, is bounded by *p*, a user specified parameter. When *p* is larger than the pairwise distance between dominant variants in the population, a subset of contigs would represent these while other contigs would capture the more distant ones. With this idea in mind, *VICUNA* outputs a set of contigs that are represented as a multiple sequence alignment (MSA) of their constituent reads. Each MSA contains substitutions and indels, which enable the capture of polymorphism in the data. Meanwhile, a single consensus sequence can be readily calculated by taking the dominant nucleotide base in each alignment column with ties broken arbitrarily. Notably, the MSAs are constructed incrementally starting from single reads to the final contigs rather than aligning reads back to the consensus post-assembly. When the viral target is known, as is the case in the examples provided in this study, a final contig representing the population mean can be built by merging contigs spanning the target region, selecting the ones that have the most read support. Nonetheless, when the target is unknown such as a microbiome sample or in cases of unknown infections, we can recover a set of contigs that represents the spectrum of the genomes in the population. This enables the identification of samples from patients co-infected with multiple viruses.

**Figure 1:**
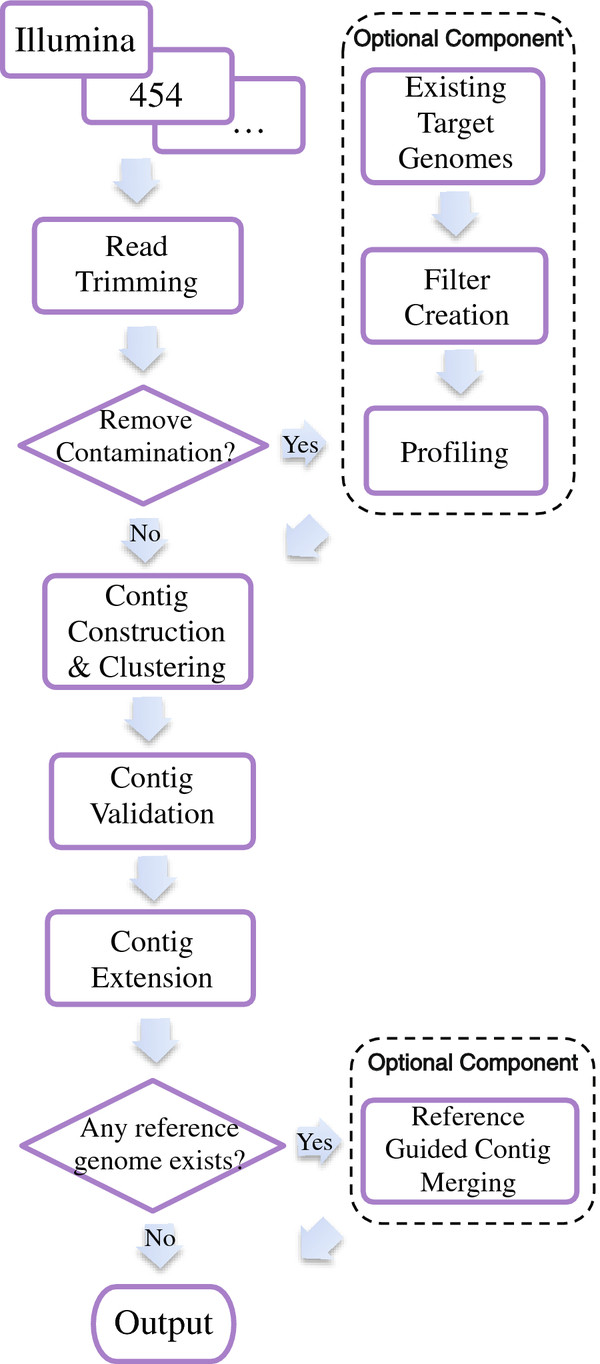
**Schematic of the *VICUNA *assembly algorithm.**

*VICUNA* consists of four key steps: read trimming, contig construction & clustering, contig validation, and contig extension & merging (Figure 1). In addition, the algorithm includes two optional modules (see Additional file 1), one to identify target and non-target reads prior to assembly, and a second to guide contig merging using a relevant reference genome if one is available.

#### Read trimming

Primer sequences or adapters may still be attached to the fragments pre-sequencing. End of reads that match any substring of primers are removed. In this step, we also remove low complexity reads that likely result from process artifacts [28].

#### Contig construction & clustering

Identifying similarities among input reads is the *ab initio* step in building contigs. To avoid the compute-expensive pairwise alignment, existing methods typically identify common *k*mers (de Bruijn graph based methods) or maximal substrings (overlap-layout-consensus based methods) among input reads, achieved using efficient data structures such as de Bruijn graphs [24,25], suffix tree/array [29], and Burrows-Wheeler transformation [30]. All use exact string matching strategy as the first pass to identify potential reads with good similarities.

To build contigs at the population level, we need to account for scenarios where diversity varies considerably from one genomic region to another, and the abundance of sequencing errors differs considerably due to highly uneven read coverage. These characteristics of the data pose the following challenges for existing methods. Exact string matching strategies may have difficulty balancing specificity vs. sensitivity in identifying similar reads: whereas a shorter *k*mer may considerably reduce specificity, sensitivity can suffer with a longer *k*mer. Also, the degree of vertices in de Bruijn graph increases considerably in both highly diversified and highly covered genomic regions, increasing the required compute resources.

To address these challenges, we adopt a min hash and an inexact string matching based similarity searching methods. The min hash is space and run-time efficient and was originally applied to cluster billions of web documents [31], and it was recently applied to DNA sequences [32]. *VICUNA* decomposes each read into *k*mers, each hashed uniformly to a large integral space (*e.g.*2^64^). The min hash value is used to represent each read. Based on the proof [31] that the similarity between any two reads, defined as the Jaccard Index, is equivalent to the probability that they have the same min hash, similar reads are held to form the same contig via common min hashes without resorting to pairwise comparison. This strategy is particularly effective in reducing read redundancy in the data. In practice, we used a two-phased min hash strategy to increase specificity (see Methods). However, this method will miss many overlaps that fail to have a common min hash, so we combine it with a seed-based approach. Using spaced seeds is proven to be more sensitive in similarity detection while maintaining the same specificity compared to searching by exact strings [33]. To identify good suffix-prefix overlaps among reads, we utilize a sliding window approach, where the window size is *w*. We then compute the subsequence of *k* (<*w*) selected bases, termed spaced-*k*mers, in each window. Sequencing errors or nucleotide polymorphism-incurred differences are neglected if they appear in the remaining (*w* − *k*) bases. Finally, reads that share any common spaced-*k*mer are held in the same contig. Unlike previous methods that store the entire spaced *k*-spectrum, we developed a strategy that keeps only a small fraction of it in the memory. Relying on the observation that a read may concurrently appear in multiple contigs, we devised an efficient partitioning algorithm to merge such contigs (see Methods).

#### Contig validation

Given *p*, the rate of variation, we would like to capture in the population, define a contig to be consist of a set of reads where each read *r* differs from the consensus of the contig by at most *p*×|*r*| bases where |*r*| denotes the read length. Abiding by this definition, we validate each contig by iteratively aligning each constituent read to the consensus, discard those that do not satisfy the criteria and update the contig consensus base calls accordingly (see Methods). This novel strategy effectively separates mis-placed reads from each contig generated in the previous step, and creates new contigs when applicable.

#### Contig extension & merging

*VICUNA* identifies contigs that share good suffix-prefix overlaps and iteratively merges them to form longer contigs until no further extension can be made. At each iteration, *VICUNA* starts with the longest contig *C*. The contig *C*^ʹ^ that shares the maximum number of paired reads with *C* is examined to identify a good suffix-prefix alignment. However, such an alignment may not be discovered due to polymorphisms captured in either contig. We resolve this issue by editing each contig before alignment. Specifically we (i) convert any consensus base that has nucleotide polymorphisms to the smallest alphabetic base, and (ii) remove non-dominant low frequency insertions. These polymorphisms will be recovered if *C* and *C*^ʹ^ were to be merged. The contig alignment is carried out by first computing a sequence of non-overlapping common substrings between the consensuses of *C* and *C*^ʹ^. We then use the Needleman-Wunsch algorithm for alignment and require the common substring blocks be directly aligned. To account for length polymorphisms that maintain the coding frame and hence represent true biological variants, any small gap comprising a multiple of three nucleotides is not penalized. Once all *de novo* merges have been completed, contigs can be further merged if mappings to a previously assembled reference genome can validate the merge.

#### Identification of target and non-target reads

Viral samples derived from clinical specimens typically contain non-target nucleic acids (*e.g.* host or other microbe DNAs and RNAs), which can comprise a large portion of the sequencing output [28]. These “contaminants” can be inherent in the sample or be artifacts that result from a sample prep method. Non-target reads increase assembly cost, memory and run-time, and can also negatively impact the quality of the assembly of the target genome. To overcome this issue, we developed an optional component (see Additional file 1) in *VICUNA* that identifies and flags such contaminant data and removes it from the assembly process. The optional module utilizes a multiple sequence alignment (MSA) comprised of available reference genomes for the target. The high degree of polymorphism in some populations compounded by errors introduced via the sample preparation process can impact the ability to identify target reads. As previously reported this situation can result in a failed alignment as a target read *r* looks too dissimilar to any known target genomic sequence [13]. To address this issue, we employ a profiling method that divides the MSA into multiple bins, and compares *k*mer compositions of *r* to each bin. If a sufficient number of similar (*i.e.* within a limited Hamming distance [34]) *k*mers spanning a sufficient length of *r* can be identified, *r* is considered to be target-alike. Moreover, we use a less stringent similarity threshold to place paired reads which could not be placed otherwise but obey the distance constraint imposed by the paired read library.

### Sequence data generation & analysis

Using previously described protocols, we amplified four large overlapping amplicons that captured the complete coding regions for DENV [35], HIV [2], and WNV [36]. Amplified products from each sample were acoustically sheared (Covaris, Woburn, MA). Indexed Illumina libraries were prepared as described [37] except that PFU Ultra II enzyme (Agilent, Santa Clara, CA) was used and 7 cycles of PCR enrichment were employed. We pooled all the samples, gel purified them (target insert size: 500-700bp), and generated 225bp paired reads using the Illumina MiSeq platform (see Methods).

**Figure 2:**
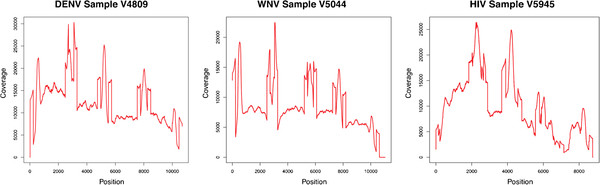
**Coverage plot.** Fold sequence coverage across the target regions of one representative sample for DENV, WNV, and HIV full-length genomes. Coverage is measured as the total number of reads uniquely aligning over a given residue; alignments are to standard references (see Methods).

The number of Illumina reads generated for each sample ranged from 0.13M to 0.95M (Additional file 1: Table S1). To evaluate the general characteristics and coverage profile of these data prior to assembly we aligned the reads to a standard reference genome for each virus (see Methods). In all cases ≥ 87.8*%* of reads were uniquely aligned to the chosen reference. As expected, alignments to the standard references were suboptimal as compared to *de novo* assemblies (Table 1 and Additional file 1: Table S1), with the WNV reference better representing the sample population than the DENV and HIV references. The WNV samples in general had a larger percentage of reads that could be uniquely aligned and a considerably smaller rate of discrepancy between the bases in the reference and the ones derived from the read alignment (Additional file 1: Table S1). The average coverage across all the samples ranged from 3,210 to 15,456 and in all cases the region of the genome targeted for sequencing was fully covered. Coverage varied across the genome within each sample; sequence coverage within an individual sample varied between the minimum and maximum by 7 - 620 fold (Figure 2 and Additional file 1: Figure S1). In general, WNV and DENV samples had greater uniformity of coverage than HIV samples, likely a result of either the greater diversity in the HIV samples or the high degree of secondary structure in the HIV genome [38].

**Table 1:** **Assembly Results for *VICUNA *, *SOAPdenovo *and *AV *454**

**Virus**	**V#**	**Method**	**# output**	**% target region**	**# contigs used for**	**% target region**	**% reads**	**non-dominant**	**# genes / total**	**run time (s) †**	**memory (G) †**
			**contigs**	**covered**	**reference**	**covered by the**	**align to**	**call rate (%)**	**with frame shift**		
			**(*≥* 350bp)**		**guided merging**	**longest contig**	**consensus**				
	V4526	*VICUNA*	10	100	1	100	95.31	0	1/11	248	0.42
		*SOAP*	19	34.51	18	4.29	16.31	17.23	-^a^	79	6.40
		*AV*454	4	100	1	100	94.69	0	5/11	507	1.10
	V4528	*VICUNA*	9	100	1	100	95.01	0	1/11	305	0.44
		*SOAP*	24	39.26	22	3.75	-^a^	-^a^	-	79	6.20
WNV		*AV*454	3	100	1	100	94.52	0	2/11	379	0.12
	V5044	*VICUNA*	8	100	1	100	95.08	0	0/11	441	0.59
		*SOAP*	32	40.23	28	3.16	8.84	17.45	-	117	6.40
		*AV*454	5	100	1	100	94.22	0.01	5/11	387	0.21
	V5048	*VICUNA*	9	100	1	100	95.08	0	0/11	212	0.43
		*SOAP*	17	24.52	15	3.49	5.67	15.90	-	90	6.40
		*AV*454	1	100	1	100	95.08	0	1/11	453	0.14
	V4809	*VICUNA*	6	100	1	100	95.64	0.01	0/11	510	0.92
		*SOAP*	40	59.7	33	3.63	16.05	17.78	-	184	6.50
		*AV*454	7	100	1	100	94.6	0.019	5/11	474	0.27
	V4813	*VICUNA*	12	100	1	100	95.12	0.01	0/11	669	1.02
		*SOAP*	49	64.33	40	3.66	18.21	18.54	-	193	6.50
DENV		*AV*454	2	100	1	100	94.18	0.04	7/11	492	0.55
	V4816	*VICUNA*	9	100	1	100	95.52	0	0/11	677	0.91
		*SOAP*	37	53.85	31	3.76	11.91	16.63	-	167	6.50
		*AV*454	5	100	2	100	94.84	0.20	5/11	471	0.32
	V4820	*VICUNA*	14	100	2	82.45	93.46	0	0/11	1158	1.20
		*SOAP*	56	70.73	46	3.62	13.37	17.20	-	234	6.50
		*AV*454	13	100	2	76.68	91.59	0.18	6/11	462	0.17
	V5937	*VICUNA*	12	100	2	93.58	93.8	0.02	0/9	516	0.86
		*SOAP*	42	48.01	30	3.95	17.72	17.71	-	142	6.40
		*AV*454	16	100	1	100	86.15	0.55	6/9	406	0.15
	V5938	*VICUNA*	18	100	1	100	93.69	0.01	0/9	281	0.62
		*SOAP*	28	40.5	25	4.16	11.33	16.08	-	111	6.40
HIV		*AV*454	15	100	1	100	88.01	0.43	5/9	443	0.21
	V5943	*VICUNA*	9	100	1	100	95.58	0.05	1/9	96.5	0.21
		*SOAP*	24	32.03	19	4.1	12.15	18.37	-	40	6.50
		*AV*454	9	97.16	1	97.16	92.52	0.80	4/9	583	0.55
	V5945	*VICUNA*	13	100	2	98.83	94.44	0.09	0/9	576	0.60
		*SOAP*	31	49.02	25	4.21	13.53	16.85	-	110	6.20
		*AV*454	7	100	2	83.32	89.54	1.18	4/9	465	0.17

^a^*SOAPdenovo* assembly is highly fragmented, a large number of short contigs were merged using the reference genome, leading to the inclusion of many low frequency variants that considerably increased the percentage of non-dominant bases found in the assembly. In the case of sample V4528, Mosaik failed to report read alignment to the consensus. The number of genes with frame shift is not measured for *SOAPdenovo*. For run time and memory, †*Soapdenovo* uses 8 threads while the other two use 1 thread. *AV*454 is run on a subset of the reads (∼ 11k, equivalent to 1% – 8% of input).

We evaluate assembly results using the following major metrics [2]: 1) the percentage of the target region captured (see Methods), 2) the percentage of reads captured by the consensus, an indicator of how closely the consensus captures the population, and 3) the *non-dominant base call rate*, defined as the percentage of consensus bases that do not represent the majority base at a given position in the read data set. In addition, we report the number of contigs that were used for reference guided merging, which indicates how “fragmented” the assembly results would be if the patient was infected with unknown viruses.

### *De novo* assembly of viral samples with less genetically diverse populations – DENV & WNV

Clinical samples derived from WNV and DENV infected hosts are typically less genetically heterogeneous compared to samples from a host harboring a chronic infection such as HIV; they are acute diseases and either provide limited time for viruses to develop mutations or undergo very rapid selection, quickly removing a majority of early variants before samples are acquired. Consensus generation in these samples is similar to haploid genome assembly, and existing assemblers are expected to work reasonably well since variants can be treated as sequencing errors and removed from the data. Nonetheless, even high quality haploid genome assemblers like *SOAPdenovo* work poorly in dealing with such data (Table 1).

*VICUNA* fully recovered the target region in all samples, and in 7 out of 8 samples the target region is captured by a single contig (Table 1) prior to guided contig merging using a reference. For WNV samples, a comparable number of reads aligned to the *VICUNA* consensus and the standard reference (Table 1 and Additional file 1: Table S1). However, *VICUNA* called the correct dominant consensus base in all cases. The *VICUNA* consensus also better represented the DENV samples; we observed an average 0.005% non-dominant call rate, and up to 2.7% more reads aligned as compared to the standard reference (Table 1 and Additional file 1: Table S1).

### *De novo* assembly of viral samples with more genetically diverse populations – HIV

HIV/AIDS is a chronic disease. As such, samples obtained from HIV infected hosts typically harbor greater genetic diversity compared to samples from acute diseases as the virus has had sufficient time to develop mutations. Given this greater genetic heterogeneity, existing short read assemblers perform poorly [2], and the use of reference genomes not derived from the sample population itself often results in a poor backbone on which to map variants.

*VICUNA* successfully assembled the full target region of genetically diverse HIV clinical samples, and captured the genome in one or two contigs (Table 1) prior to guided contig merging using a reference. Compared to the standard reference genome, on average over 4% more reads can be aligned to *VICUNA* consensus; we observed an average 0.043% non-dominant call rate (Table 1 and Additional file 1: Table S1).

### Comparing *VICUNA* assembly results to other assemblers

We compare the performance of *VICUNA* with existing *de novo* genome assemblers *SOAPdenovo*[24] and *AV *454 [2]; *VICUNA* outperformed both. We report the result of *SOAPdenovo*, which is a representative for de Bruijn graph based short read assemblers and its result is comparable to several of other assemblers of the same kind, such as MetaVelvet [39], used for metagenomic data sets, and Velvet Columbus [22], used for reference assisted assembly. *AV *454 is selected as a representative for overlap-layout-consensus based assemblers, as it was designed specifically for viral population consensus assembly. The *AV *454 algorithm was originally designed for 454 read data sets, and for application here we optimized it to assemble paired Illumina reads (see Additional file 1). Because *AV *454’s performance deteriorates as the coverage increases beyond a certain threshold, we down-sampled each dataset by randomly selecting about 5,500 read pairs for each sample. Cortex [13] was recently developed for assembling diploid genomes or a mixed population using color-coded de Bruijn graphs. However, it appears not to be suitable for viral population assembly as when applied to current samples, it generated an excessive number of contigs, which cannot be reasonably handled.

*SOAPdenovo* generated a large number of contigs, but only a small percentage of them exceed the minimum length cutoff of 350bp (Table 1). In total, these contigs account for less than 5% of the target region in each sample. Since the algorithm is not able to utilize sufficient reads to form contigs, the dominant base call rate was < 85*%*. *SOAPdenovo*’s performance was not markedly better at assembling acute versus chronic viruses.

*AV *454 outperformed *SOAPdenovo* for all viruses, but was inferior to *VICUNA*. *VICUNA* has the capacity to scale to tens of thousands fold coverage, whereas, *AV *454 is limited to a few hundred fold coverage. This may significantly affect the performance of *AV *454 in clinical samples rich in contamination (e.g. > 99% contaminants), where the down-sampling required by *AV *454 would result in insufficient coverage of target genome [28]. In all samples, *VICUNA* has a better accuracy in producing intact genes that have no frame shift (Table 1). For WNV samples, the dominant base accuracy was similar, but on average 0.5% fewer reads align to *AV *454 consensus than to *VICUNA* consensus (Table 1). For DENV samples V4816 and V4820, the *VICUNA* consensus was significantly more accurate in dominant base calls (*p*-value < 2 × 10^−5^; *χ*^2^ test), and approximately 1-2% more reads aligns to it as compared to the *AV *454 consensus. For HIV samples, where the genetic total diversity is higher, the advantage of *VICUNA* is more pronounced. Up to 6% more reads can be aligned to *VICUNA* consensus compared to *AV *454 consensus, and it is over 100 times more accurate (*p*-value < 1 × 10^−8^; *χ*^2^ test) in dominant base calling.

### *VICUNA* is able to capture low frequency length polymorphisms and large deletions in viral samples

Other than nucleotide polymorphisms, length polymorphisms are frequently observed in viral samples, exemplified by a small number of nucleotide insertions at certain genomic loci in a subset (typically a small fraction) of viral genomes in the population. With the pressure to preserve coding frames, these insertions typically consist of a multiple of three bases. *VICUNA* is capable of capturing non-dominant insertions that occur at the frequency of at least *p*, a user specified parameter, when compared to the dominant variant at the same loci. In viral samples, we are interested in *p* ≤ 5*%*, which are typically treated as sequencing errors in haploid genome assembly. When *p*=5*%* , we observed in WNV, DENV, and HIV samples, 0.003%, 0%, and 0.082% low frequency insertions, respectively, which fits the expectation that HIV genomes have a larger amount of length polymorphisms compared to the other two viruses.

Large deletions have been observed previously in viral population studies (*e.g.*[36,40]). Since there is no clear definition on the minimum size, we recorded all deletions ≥ 500*bp* in length (Additional file 1: Table S2). We observed large deletions in two DENV and all four HIV samples and, in some of them, we found multiple types of variants. The majority of these deletions occur in the Env and Pol genes in HIV and in the NS3 gene in DENV. These large deletions may be real biological mutants or they could be amplification or sequencing artifacts (*e.g.* chimeras). To eliminate the possibility that our observations are due to Illumina specific artifacts, we validated the observed deletions using 454 read data sets from the same samples. Almost all large deletions in HIV samples are observed with the exact breakpoints in the 454 data (Additional file 1: Table S2), while none of the DENV deletions were observed. These results suggest that *de novo* assembly of Illumina reads via *VICUNA* provides the ability to observe novel large deletion events.

### *VICUNA* is applicable to other data types

Many existing sequence data sets that characterize viral populations were generated using the Roche 454 technology. To demonstrate the applicability of *VICUNA* to such data, we used *VICUNA* to generate a consensus for WNV, DENV, and HIV clinical samples sequenced by 454. In all cases, the full target region is recovered with high fidelity (Additional file 1: Table S3). *VICUNA* achieves a 100% dominant call rate. Since reads generated by the Life Sciences Ion Torrent technology share similar error properties to 454 reads, we anticipate that *VICUNA* will be suitable for assembly of these data as well.

## Conclusions

We presented *VICUNA*, a program for *de novo* consensus assembly of genetically heterogeneous viral populations. For each read data, *VICUNA* outputs consensus sequence(s) and for each consensus sequence, the multiple sequence alignment of its constituent reads. We have demonstrated that *VICUNA* recovers consensus genomes with high fidelity for viral intra-host samples obtained from patients infected with either acute or chronic diseases and that it outperforms other available assemblers in both the accuracy and continuity of the consensus genome. The ability to assemble *de novo* accurate and contiguous consensus genomes from ultra-deep short read sequence data derived from the Illumina platform or other comparable technologies provides the ability to capture a higher resolution map of viral genetic variants in an infected host with greater cost efficiency than previously possible. The availability of a consensus derived from the sample itself provides greater accuracy with respect to aligning reads prior to variant detection and as such *VICUNA* will enable improved detection of low frequency variants and subsequent haplotype identification in genetically heterogeneous populations. While *VICUNA* was developed for the analysis of viral populations its application to other heterogeneous sequence data sets such as metagenomic or tumor cell population samples may prove beneficial in these fields of research.

## Methods

### Illumina MiSeq platform configuration

To increase the read length on the Illumina MiSeq platform, the configuration files were modified to increase signal intensity as the read progresses; this helps to ensure fidelity of clusters. Specifically, Exposure was set to 342 for bases A/C and 165 for bases T/G. Ramp values were increased to 4.8 for bases A/C and to 2.3 for bases T/G. LEDSnapCurrentMA value was changed to 1500.

### Read alignment to reference genomes

The reference genomes chosen for aligning the input reads are NC001475 (NCBI accession number) for Dengue, NC009942 for West Nile, and a modified version of the HXB2 reference K03455 for HIV. The HIV reference genome is a subsequence of HXB2 (from 779bp to 9551bp) and is corrected for a frame-shift and a premature stop codon (available upon request). The target regions for analyses consist of the complete CDS of each reference genome, which corresponds to positions 95-10267 for Dengue, 97-10398 for West Nile and 12-8638 for HIV.

Read alignment to reference genome or assemblies are carried out using Mosaik v1.1.0013 ( http://bioinformatics.bc.edu/marthlab/Mosaik), with parameter setting “*st* = illumina, *hs* = 10, *act* = 15, *bw* = 29, *mmp* = 0.25 and *minp* = 0.25”.

### General definitions, notations, and techniques

Given two set of integers *S*_1_ and *S*_2_, the similarity measure *Jaccard Index* between them is given by $\frac{\left|S_{1}\cap S_{2}\right|}{\left|S_{1}\cup S_{2}\right|}$. The *k*-spectrum (all possible *k*mers) of a DNA sequence *r*, including both forward and reverse complementary strands, is denoted as *r*^*k*^. Let *hd*(*r*_1_, *r*_2_) denote the Hamming distance between DNA sequences *r*_1_ and *r*_2_(|*r*_1_ |=| *r*_2_ |), then the *d*-neighbors of *r*_1_ in *S* is given by $\left\{r^{\prime }|(\mathrm{hd}(r_{1},r^{\prime })\leq d)\wedge (|r|=|r^{\prime }|)\wedge (r^{\prime }\in S)\right\}$. The substring from position *i* to *j* of *r* is denoted as *r*[*i*,*j*].

We index each read by a 2-tuple 〈 *id*(file),offset 〉, where the offset is the start position of the read in the file. A read can be retrieved from hard drive using its index. Reads are loaded into memory from hard drive in batches, where the batch size is a user specified parameter.

Define a function *id*(*x*) to return a unique identifier of *x*, let it be a file, a read, or a contig. We represent each contig *C* in the form of a multiple sequence alignment of its constituent reads. The alignment encoding of *r*_*i*_ is given by a 5-tuple *a*_*i*_ = 〈 *id*(*r*_*i*_), |*r*_*i*_|,*d*_*i*_,*I*_*i*_,**g** 〉, where *d*_*i*_ is the start position of *r*_*i*_ on *C*, *I*_*i*_ indicates if *r*_*i*_ belongs to the forward (*I*_*i*_ = 1) or reverse complementary (*I*_*i*_ = 0) strand of *C*, and **g** specifies insertions in *r*_*i*_. Each insertion is denoted as a 2-tuple, specifying its start position on *r*_*i*_ and the size of insertion. Using this representation, the dominant base in each alignment column is considered as the consensus base. Further, we define the *layout* of *C* to be an ordering of reads (*r*_1_, *r*_2_, …,*r*_*i*_, …) such that *d*_*i*_ ≤ *d*_*i* + 1_. A *low frequency non-dominant fragment* of *C* is a subsequence of *C* with significantly lower coverage (a pre-specified value) compared to flanking regions. A *polymorphic site* in *C* is a position where more than one valid type of nucleotides occur in the alignment.

### *VICUNA* algorithm

#### Contig construction

Initially, two min hash values are generated for each read, one for the forward, and the other for the reverse complementary strand. The min hash of the forward strand of read *r* is calculated as follows. First, compute the hash value of each *k*mer in the forward strand. After sorting, we obtain an integer sequence (*h*_1_, *h*_2_, …, *h*_|*r*| − *k* + 1_), where *h*_*i*_ ≤ *h*_*i* + 1_. In this order, *h*_*i*_s are converted to string representation and concatenated to form a new string *S*. Apply the same procedures to *S* as to the forward strand of *r*: decompose *S* to *k*-substrings, hash, then sort. The min hash is the first integer in the sorted sequence. The same strategy is applied to the reverse complementary strand of *r*. This way, two hash values (integers) are generated to represent every read in the input data. Two reads sharing any min hash value have a high probability to be near identical, albeit a small edit distance. Clustering reads based on common min hash values avoids expensive pairwise comparison. Next, we transform each read cluster to a contig by first identifying a *k* mer that is shared among all reads, then generate alignment of these reads by directly matching this *k* mer. Min hash technique may not necessarily lead to the discovery of all good suffix-prefix overlaps among reads. We further employ spaced seed based similarity search strategy to initialize new contigs or add homologous reads to existing contigs. The concept of a *k*mer can be generalized to denote a concatenation of *k* selected bases from a substring with length *w* for *w* ≥ *k*. In the following, we require *w* >*k*. $r_{i}^{k}$ is generated by sliding a window of length *w* across *r*_*i*_and its reverse complementary strand, and *k* fixed positions are selected and concatenated to form a *k* mer. The template that specifies which positions to be selected is termed a *spaced seed*. We use common *k* mers as the seeds to identify good read similarity. Rather than recording all possible *k*mers present in the dataset, which is memory demanding when *k* is large, we maintain a database **D**, initialized to be empty. An entry in **D**is a 2-tuple 〈 *x*,(*id*(*r*),*p*) 〉, where the key *x* denotes a *k* mer and the value is a pair with *p* denoting the start position of *x* on *r*. **D** is populated by including $r_{i}^{k}$ if none of the *k* mers in $r_{i}^{k}$ is already included in **D**. If there exists a read *r*^ʹ^ that shares a common *k*mer with *r*_*i*_, then we add *r*_*i*_ to contig *C*^ʹ^ where *r*^ʹ^ resides. Since the *k*mer positions in both *r*_*i*_ and *r*^ʹ^ are known as well as the relative position of *r*^ʹ^ in *C*^ʹ^, the addition of *r*_*i*_ to *C*^ʹ^ takes constant time.

The above strategies are outlined in Additional file 1: Algorithm 1 and a simplified example is given in Additional file 1: Figure S2.

#### Contig clustering

Thus far, a read can occur in multiple contigs, which are to be merged. In the context of graph theory, let each contig be a vertex and two vertices are connected by an edge if they share some common read. The goal is to then identify connected components of this graph, each representing a new contig after merging. Union-find algorithm is typically used for such a purpose. When applied to current application, we can obtain a solution in *O* (*n*^2^) time, where *n* is the number of input reads and assuming to merge two contigs takes constant time. As in the worst scenario, the total number of vertices in the graph is *O*(*n*^2^). Instead, we devise a graph partitioning strategy, presented in Additional file 1: Algorithm 2, which reduces run time to *O*(*n* log *n*). Initially, contigs are sorted in a decreasing order with respect to the number of reads they contain. The graph is iteratively partitioned to be non-overlapping stars. The center of each star is chosen to be the contig that was not yet contained by other stars but has the largest number of reads, and all its neighbors become leaves of the star. After partitioning, all contigs in each star are merged to form a new contig. These newly formed contigs will participate in the next round of merging until the partition of contigs also corresponds to a partition of input reads. The number of iterations for graph partitioning is bounded by *O*(log *n*) since each iteration at least halves the number of vertices. Given a star, each leaf contig shares a common read with the center contig. During the merging, the common read guides a direct alignment of different contigs, which takes linear time in the total number of reads involved in the star. At a given iteration, since no stars share common reads, the time complexity to generate merged contigs for all stars is *O*(*n*). Therefore, the overall clustering step takes *O*(*n* log *n*) time.

#### Contig validation

Reads sampled from different genomic locations may be misplaced in the same contig either due to hash collisions (contig construction stage) that enable dissimilar reads to share the same min hash value; or because of the graph partitioning strategy (contig clustering stage) employed to merge contigs via common reads. We set the following two goals for validating a contig: 1) Put an explicit distance constraint *max*_*d*_ between the consensus of each contig and each of its constituent read. *max*_*d*_ corresponds to an upper bound of the diversity (or polymorphisms) we would like to capture in the sample. 2) Split potential chimeric contigs. As illustrated in Additional file 1: Figure S3, a chimeric contig may be formed either due to the presence of short homologous regions on the reference genome (a), or due to chimeric reads resulted as sequencing artifact (b).

Contig validation procedure is presented in Additional file 1: Algorithm 3. First, for each contig *C*, a consensus is generated and is compared against every constituent read *r*. *r* is retained only if the edit distance between *r* and the consensus is within *d*. Otherwise, it is set aside in *C*_*rem*_ for further consideration. The consensus of *C* is updated whenever a read is removed, which may result in a re-comparison between the updated consensus with each of the constituent read. Updates stop when no changes were made. The same procedure is applied to *C*_*rem*_. To achieve the second goal, the two types of chimeric contigs are resolved differently. The first type is resolved by requiring adjacent reads in the *layout* of the contig to have a minimum overlap of *min*_*ol*_. For the second type, we use the observation that chimeric reads typically occur less frequently compared to the number of non-chimeric reads sampled from the two corresponding genomic regions. This corresponds to observing a much smaller number of reads colored both red and blue than the number of reads colored red or blue alone (Additional file 1: Figure S3 (b)). Formally, for each read *r*, consider only the reads that have sufficient overlap (≥ *min*_*ol*_) with *r* in the layout of the contig, and let *n*_*b*_ and *n*_*a*_ be the number of reads before and after *r*, respectively. Break the contig after any read, where $\frac{n_{b}}{n_{a}}(\geq \mathrm{ma}x_{\mathrm{rt}})$ is a local maximal. The input parameter *max*_*rt*_ specifies the ratio in coverage change that is likely due to a chimeric formation. Although the attempt to break chimeric contigs may also lead to the split of valid contigs, they will be considered later.

#### Contig extension

Validated contigs are extended to form longer ones when good suffix-prefix overlaps can be identified among them (Additional file 1: Algorithm 4). These contigs are sorted in the order of decreasing length and considered in that order. For the current longest contig *C*_*l*_, all its neighboring contigs, defined as ones that are linked to *C*_*l*_ by paired reads, are identified and stored in **N**. Then, **N** is sorted in an increasing order of the number of links shared with *C*_*l*_. Define a *delegate* of contig *C* to be a sequence derived from the consensus of C by 1) removing low frequency non-dominant fragments, and 2) converting the consensus base in each polymorphic site to be the alphabetically smallest one. We then compute the delegates for *C*_*l*_ and each contig in **N**. The delegate of *C*_*l*_ is compared with each delegate of contig in **N** to identify good suffix-prefix overlap (Additional file 1: Figure S4 and Additional file 1: Algorithm 5): Given two sequences *s*_0_ and *s*_1_, first, identify a sequence of non-overlapping common substrings between them relying on common *k* mers, stored in a hash table. The coordinates of all substrings so identified are stored in a vector **V**. Needleman-Wunsch algorithm is then applied to align *s*_0_ and *s*_1_ by forcing the common substring blocks to be directly aligned. To capture length polymorphisms, a gap in the alignment is not penalized if its length is less or equal than a user specified threshold and is divisible by three (to avoid penalizing length polymorphisms that preserve coding frame). A *valid* alignment satisfies the following criteria: 1) the similarity between aligned region is ≥ *min*_*s*_, 2) the length of the aligned region is ≥ *min*_*ol*_, and 3) the overhang of the alignment is ≤ *max*_*oh*_. The first two parameters constrain the quality of alignment while the third parameter loosens the constraint to account for insufficient trimming or bad quality bases in the end of contigs, which are likely sequencing errors. Note that there may exist multiple valid alignments between two sequences, where we take the first one that is found to be valid.

When a valid alignment exists between *C*_*l*_ and a contig *C*_*r*_ ∈ **N**, they are merged to form a longer contig. Since a contig is represented as the MSA of its constituent reads (recall from section “General definitions, notations, and techniques”), the merged contig consists of all reads belonging to *C*_*l*_ and *C*_*r*_. The start position of each read in the new contig is adjusted to its distance with respect to the beginning of the resulting alignment; any indel that was introduced in the consensus of *C*_*l*_ or *C*_*r*_ as the result of alignment was included in the corresponding reads that overlap with the indel. The time complexity of merging two contigs post-alignment, *i.e.* to merge two existing MSAs, is linear to the number of reads involved.

Previously assembled viral genomes of the same type provide valuable information to further improve the assembly if we have not yet generated any single consensus that represent the full length genome. By aligning contigs to the selected reference, we can further merge any pair of them if they overlap on the reference.

## Competing interests

The authors declare that they have no competing interests.

## Authors contributions

XY & MCZ conceived and designed the algorithm; XY implemented VICUNA; PC & SG provided supporting code; NJL, JZL, JQ & EMR optimized laboratory protocols for Illumina sequencing; XY, PC, SG, MCZ & MRH analyzed data; XY, PC, MCZ & MRH wrote the manuscript with input from all authors; MCZ and MRH contributed equally to this paper. All authors read and approved the final manuscript.

## Supplementary Material

Additional file 1 **Supplementary Materials.** Supplementary materials include pseudo code of all algorithms, supplementary figures and tables. Click here for file

## References

[B1] Vignuzzi M Stone JK Andino R Ribavirin and lethal mutagenesis of poliovirus: molecular mechanisms, resistance and biological implications Virus res 2005 107 2 173 181 10.1016/j.virusres.2004.11.007 15649563

[B2] Henn MR Boutwell CL Charlebois P Lennon NJ Power KA Macalalad AR Berlin AM Malboeuf CM Ryan EM Gnerre S Zody MC Erlich RL Green LM Berical A Wang Y Casali M Streeck H Bloom AK Dudek T Tully D Newman R Axten KL Gladden AD Battis L Kemper M Zeng Q Shea TP Gujja S Zedlack C Gasser O Whole Genome Deep Sequencing of HIV-1 Reveals the Impact of Early Minor Variants Upon Immune Recognition During Acute Infection PLoS Pathogens 2012 8 3 e1002529 10.1371/journal.ppat.1002529 22412369 PMC3297584

[B3] Herbeck JT Rolland M Liu Y McLaughlin S McNevin J Zhao H Wong K Stoddard JN Raugi D Sorensen S Genowati I Birditt B McKay A Diem K Maust BS Deng W Collier AC Stekler JD McElrath MJ Mullins JI Demographic processes affect HIV-1 evolution in primary infection before the onset of selective processes J Virol 2011 85 15 7523 7534 10.1128/JVI.02697-10 21593162 PMC3147913

[B4] Lauck M Alvarado-Mora MV Becker EA Bhattacharya D Striker R Hughes AL Carrilho FJ O’Connor DH Pinho JR Analysis of Hepatitis C Virus Intrahost Diversity across the Coding Region by Ultradeep Pyrosequencing J Virol 2012 86 7 3952 3960 10.1128/JVI.06627-11 22278255 PMC3302523

[B5] Jerzak G Bernard KA Kramer LD Ebel GD Genetic variation in West Nile virus from naturally infected mosquitoes and birds suggests quasispecies structure and strong purifying selection J Gen Virol 2005 86 Pt 8 2175 2183 16033965 10.1099/vir.0.81015-0 PMC2440486

[B6] Murcia PR Baillie GJ Daly J Elton D Jervis C Mumford JA Newton R Parrish CR Hoelzer K Dougan G Parkhill J Lennard N Ormond D Moule S Whitwham A McCauley JW McKinley TJ Holmes EC Grenfell BT Wood JL Intra- and interhost evolutionary dynamics of equine influenza virus J Virol 2010 84 14 6943 6954 10.1128/JVI.00112-10 20444896 PMC2898244

[B7] Vignuzzi M Stone JK Arnold JJ Cameron CE Andino R Quasispecies diversity determines pathogenesis through cooperative interactions in a viral population Nature 2006 439 7074 344 348 10.1038/nature04388 16327776 PMC1569948

[B8] Lin SR Hsieh SC Yueh YY Lin TH Chao DY Chen WJ King CC Wang WK Study of sequence variation of dengue type 3 virus in naturally infected mosquitoes and human hosts: implications for transmission and evolution J Virol 2004 78 22 12717 12721 10.1128/JVI.78.22.12717-12721.2004 15507664 PMC525091

[B9] Thai KT Henn MR Zody MC Tricou V Nguyet NM Charlebois P Lennon NJ Green L de Vries P J Hien TT Farrar J van Doorn H R de Jong M D Birren BW Holmes EC Simmons CP High-resolution analysis of intrahost genetic diversity in dengue virus serotype 1 infection identifies mixed infections J Virol 2012 86 2 835 843 10.1128/JVI.05985-11 22090119 PMC3255838

[B10] Macalalad AR Zody MC Charlebois P Lennon NJ Newman RM Malboeuf CM Ryan EM Boutwell CL Power KA Brackney DE Pesko KN Levin JZ Ebel GD Allen TM Birren BW Henn MR Highly sensitive and specific detection of rare variants in mixed viral populations from massively parallel sequence data PLoS Comput Biol 2012 8 3 e1002417 [ http://dx.doi.org/10.1371/journal.pcbi.1002417] 10.1371/journal.pcbi.1002417 22438797 PMC3305335

[B11] Koboldt DC Chen K Wylie T Larson DE McLellan MD Mardis ER Weinstock GM Wilson RK Ding L VarScan: variant detection in massively parallel sequencing of individual and pooled samples Bioinformatics 2009 25 17 2283 2285 [ http://dx.doi.org/10.1093/bioinformatics/btp373] 10.1093/bioinformatics/btp373 19542151 PMC2734323

[B12] McKenna A Hanna M Banks E Sivachenko A Cibulskis K Kernytsky A Garimella K Altshuler D Gabriel S Daly M DePristo MA The Genome Analysis Toolkit: a MapReduce framework for analyzing next-generation DNA sequencing data Genome Res 2010 20 9 1297 1303 [ http://dx.doi.org/10.1101/gr.107524.110] 10.1101/gr.107524.110 20644199 PMC2928508

[B13] Iqbal Z Caccamo M Turner I Flicek P McVean G De novo assembly and genotyping of variants using colored de Bruijn graphs Nat Genet 2012 44 2 226 232 [ doi:10.1038/ng.1028] 10.1038/ng.1028 22231483 PMC3272472

[B14] Archer J Rambaut A Taillon BE Harrigan PR Lewis M Robertson DL The evolutionary analysis of emerging low frequency HIV-1 CXCR4 using variants through time–an ultra-deep approach PLoS Comput Biol 2010 6 12 e1001022 10.1371/journal.pcbi.1001022 21187908 PMC3002995

[B15] Willerth SM Pedro HA Pachter L Humeau LM Arkin AP Schaffer DV Development of a low bias method for characterizing viral populations using next generation sequencing technology PloS one 2010 5 10 e13564 10.1371/journal.pone.0013564 21042592 PMC2962647

[B16] Eriksson N Pachter L Mitsuya Y Rhee SY Wang C Gharizadeh B Ronaghi M Shafer RW Beerenwinkel N Viral population estimation using pyrosequencing PLoS Comput Biol 2008 4 4 e1000074 18437230 10.1371/journal.pcbi.1000074 PMC2323617

[B17] Palacios G Druce J Du L Tran T Birch C Briese T Conlan S Quan PL Hui J Marshall J Simons JF Egholm M Paddock CD Shieh WJ Goldsmith CS Zaki SR Catton M Lipkin WI A new arenavirus in a cluster of fatal transplant-associated diseases N Engl J Med 2008 358 10 991 998 10.1056/NEJMoa073785 18256387

[B18] Yozwiak NL Skewes-Cox P Stenglein MD Balmaseda A Harris E DeRisi JL Virus identification in unknown tropical febrile illness cases using deep sequencing PLoS Negl Trop Dis 2012 6 2 e1485 [ http://dx.doi.org/10.1371/journal.pntd.0001485] 10.1371/journal.pntd.0001485 22347512 PMC3274504

[B19] Myers EW Sutton GG Delcher AL Dew IM Fasulo DP Flanigan MJ Kravitz SA Mobarry CM Reinert KH Remington KA Anson EL Bolanos RA Chou HH Jordan CM Halpern AL Lonardi S Beasley EM Brandon RC Chen L Dunn PJ Lai Z Liang Y Nusskern DR Zhan M Zhang Q Zheng X Rubin GM Adams MD Venter JC A whole-genome assembly of Drosophila Science 2000 287 5461 2196 2204 10.1126/science.287.5461.2196 10731133

[B20] Batzoglou S Jaffe DB Stanley K Butler J Gnerre S Mauceli E Berger B Mesirov JP Lander ES ARACHNE: a whole-genome shotgun assembler Genome Res 2002 12 177 189 10.1101/gr.208902 11779843 PMC155255

[B21] Huang X Wang J Aluru S Yang SP Hillier L PCAP: a whole-genome assembly program Genome Res 2003 13 9 2164 2170 10.1101/gr.1390403 12952883 PMC403719

[B22] Zerbino DR Birney E Velvet: algorithms for de novo short read assembly using de Bruijn graphs Genome Res 2008 18 5 821 829 10.1101/gr.074492.107 18349386 PMC2336801

[B23] Simpson JT Wong K Jackman SD Schein JE Jones SJM Birol I ABySS: a parallel assembler for short read sequence data Genome Res 2009 19 6 1117 1123 10.1101/gr.089532.108 19251739 PMC2694472

[B24] Li H Homer N A survey of sequence alignment algorithms for next-generation sequencing Brief Bioinform 2010 11 5 473 483 10.1093/bib/bbq015 20460430 PMC2943993

[B25] Gnerre S Maccallum I Przybylski D Ribeiro FJ Burton JN Walker BJ Sharpe T Hall G Shea TP Sykes S Berlin AM Aird D Costello M Daza R Williams L Nicol R Gnirke A Nusbaum C Lander ES Jaffe DB High-quality draft assemblies of mammalian genomes from massively parallel sequence data Proc Natl Acad Sci USA 2011 108 4 1513 1518 10.1073/pnas.1017351108 21187386 PMC3029755

[B26] Grabherr MG Haas BJ Yassour M Levin JZ Thompson DA Amit I Adiconis X Fan L Raychowdhury R Zeng Q Chen Z Mauceli E Hacohen N Gnirke A Rhind N di Palma F Birren BW Nusbaum C Lindblad-Toh K Friedman N Regev A Full-length transcriptome assembly from RNA-Seq data without a reference genome Nat Biotechnol 2011 29 7 644 652 10.1038/nbt.1883 21572440 PMC3571712

[B27] Pevzner PA Tang H Waterman MS An Eulerian path approach to DNA fragment assembly Proc Natl Acad Sci USA 2001 98 17 9748 9753 10.1073/pnas.171285098 11504945 PMC55524

[B28] Malboeuf CM Yang X Charlebois P Berlin A Qu J Casali M Ryan E Boutwell CL Pesko K Power K Lennon NJ Allen TM Ebel GD Zody M Henn MR Levin JZ Complete viral genome sequencing of ultra-low viral copy samples by sequence-independent amplification Nucleic Acids Res 2012 doi:10.1093/nar/gks794 10.1093/nar/gks794 PMC3592391 22962364

[B29] Kalyanaraman A Emrich S Schnable P Aluru S Assembling genomes on large-scale parallel computers J Parallel and Distributed Comput 2007 67 12 1240 1255 10.1016/j.jpdc.2007.05.014

[B30] Simpson JT Durbin R Efficient construction of an assembly string graph using the FM-index Bioinformatics 2010 26 12 i367 i373 10.1093/bioinformatics/btq217 20529929 PMC2881401

[B31] Broder AZ Glassman SC Manasse MS Zweig G Syntactic clustering of the web Comput Netw ISDN Syst 1997 29 1157 1166 10.1016/S0169-7552(97)00031-7

[B32] Yang X Zola J Aluru S Parallel Metagenomic Sequence Clustering Via Sketching and Maximal Quasi-clique Enumeration on Map-Reduce Clouds 2011 IEEE Computer Society, Washington 1223 1233

[B33] Li M Ma B Zhang L Superiority and complexity of the spaced seeds Proceedings of the seventeenth annual ACM-SIAM symposium on Discrete algorithm, SODA ’06 2006 ACM, New York 444 453

[B34] Hamming RW Error detecting and error correcting codes Bell Syst Tech J 1950 29 2 147 160

[B35] Parameswaran P Charlebois P Tellez Y Nunez A Ryan E Malboeuf C Levin J Lennon N Balmaseda A Harris E Henn M Genome-wide patterns of intra-human dengue virus diversity reveal associations with viral genetics and inter-host diversity J Virol 2012 86 16 8546 58 10.1128/JVI.00736-12 22647702 PMC3421746

[B36] Pesko KN Fitzpatrick KA Ryan EM Shi PY Zhang B Lennon NJ Newman RM Henn MR Ebel GD Internally deleted WNV genomes isolated from exotic birds in New Mexico: Function in cells, mosquitoes, and mice Virology 2012 427 10 17 10.1016/j.virol.2012.01.028 22365325 PMC3312038

[B37] Levin JZ Yassour M Adiconis X Nusbaum C Thompson DA Friedman N Gnirke A Regev A Comprehensive comparative analysis of strand-specific RNA sequencing methods Nat methods 2010 7 9 709 715 10.1038/nmeth.1491 20711195 PMC3005310

[B38] Watts JM Dang KK Gorelick RJ Leonard CW Bess JJW Swanstrom R Burch CL Weeks KM Architecture and secondary structure of an entire HIV-1 RNA genome Nature 2009 460 7256 711 716 10.1038/nature08237 19661910 PMC2724670

[B39] Namiki T Hachiya T Tanaka H Sakakibara Y MetaVelvet : An extension of Velvet assembler to de novo metagenome assembly from short sequence reads Nucleic Acids Res 2012 doi:10.1093/nar/gks678 10.1093/nar/gks678 PMC3488206 22821567

[B40] Salazar-Gonzalez JF Salazar MG Keele BF Learn GH Giorgi EE Li H Decker JM Wang S Baalwa J Kraus MH Parrish NF Shaw KS Guffey MB Bar KJ Davis KL Ochsenbauer-Jambor C Kappes JC Saag MS Cohen MS Mulenga J Derdeyn CA Allen S Hunter E Markowitz M Hraber P Perelson AS Bhattacharya T Haynes BF Korber BT Hahn BH Shaw GM Genetic identity, biological phenotype, and evolutionary pathways of transmitted/founder viruses in acute and early HIV-1 infection J Exp Med 2009 206 6 1273 1289 10.1084/jem.20090378 19487424 PMC2715054

