# Supplementary material for: De novo assembly of highly diverse viral populations
Source: BMC Genomics. 2012 Sep 13;13:475. doi: 10.1186/1471-2164-13-475 (PMC3469330; doi:10.1186/1471-2164-13-475)
Supplement: Additional file 1 — Supplementary Materials. Supplementary materials include pseudo code of all algorithms, supplementary figures and tables. [file 1471-2164-13-475-S1.pdf]

# Supplementary Materials

**Parameter setting for assembly programs.** *SOAPdenovo* (v1.05) is used in our experiments. For viral genome assembly, increasing the length of $k$mer used by *SOAPdenovo* will significantly increase the specificity, but its ability to capture diversity in the data will decrease. Since it is unclear which $k$ value will yield the best performance, we tested three different values 23, 51, 99, respectively for each dataset. The results are comparable regardless of the values we are using, hence, in the comparison, we reported the result for $k = 23$.

The version 39605 of the Arachne package was used when running *AV*454. An additional component was created (available on request) to facilitate Arachne processing of paired Illumina reads with a parameter "*cov*=250", indicating a downsampling of reads such that an average of 250x genome coverage is achieved. *AV*454 module was run with the option "PIPELINE=paired" to handle Illumina paired reads, and the genome size is set as "GSIZE=10000". Other parameters were left to default settings.

The default parameter settings are used for all samples when running *VICUNA*.

**Filter creation for target-alike reads.** We illustrated our method by creating a filter for HIV1B. The same method can be applied to other viral genomes. 1057 available full length HIV1B genomes were obtained from the LANL database (`http://www.hiv.lanl.gov/`), representing a wide range of genome diversity. These sequences were directly aligned using MUSCLE [1], resulting in a multiple sequence alignment (MSA) of length 21679. This is over the twice the length of the standard HIV genome size (∼10kbp). 17 sequences that create single large insertions in the alignment were removed, since they were likely misassembled. The remaining sequences were re-aligned, resulting in an alignment with a more reasonable length of 14232. We can further remove spurious genomes and re-align the remaining ones until satisfactory. The final alignment serves as the filter. Note that to create MSA for a large number of sequences is compute intensive, and is less accurate as the number of sequences increases. However, we do not require the filter to be free of mis-assembled sequences, nor do we require the filter to include all previously assembled genomes.

**Profiling.** The MSA filter renders each genome equivalent in length by introducing gaps in the alignment. We divide the MSA into bins, each specified by a 2-tuple $b_i = \langle s_i, e_i \rangle$, where $s_i$ ($e_i$) denotes the start (end) position of the $i^{th}$ ($0 \leq i < n_b$, $n_b$ is a user specified parameter) bin on the MSA. To calculate $b_i$, first identify the longest genome $G$, and assign $|G|/n_b$ bases to each bin. Then $b_i = \langle |G| * i/n_b, |G| * (i+1)/n_b \rangle$ for $0 \leq i \leq n_b - 1$ if $G$ contains no gaps. Otherwise, $b_i$ is adjusted to include gaps. $b_i$ determines the subsequence of each genome that belongs to the $i^{th}$ bin, where the $k$-spectrum is then calculated. To account for the case that a read may overlap with two adjacent bins, we include any $k$mer that overlaps with position $s_i$ in the $k$-spectrum of $b_i$. In addition, low frequency $k$mers are removed from consideration.

Procedure for assigning read $r$ to bins. 1) For every $k$mer $x$ in $r^k$, assign it to the $i^{th}$ bin if its $k$-spectrum contains a $d$-neighbor of $x$. Note, $x$ can be assigned to multiple bins. 2) Consider all $k$mers in $r^k$ that were assigned to the $i^{th}$ bin, if the total number of positions covered by these $k$mers in $r$ divided by $|r|$ is above a given threshold $t$, the $i^{th}$ bin is held as a candidate for $r$ to be assigned. To account for the case when $r$ spans two bins, we consider $k$mers in $r^k$ that were assigned to adjacent bins. 3) Consider the paired reads $(r, r')$: assign both concurrently to bins that obey the maximum distance constraint of the paired read library size. Otherwise, we use a more stringent threshold $t'$ ($> t$) to assign them individually.

## References


1. Edgar, R. C. Muscle: multiple sequence alignment with high accuracy and high throughput. *Nucleic Acids Res* **32**(5), 1792–7 (2004).

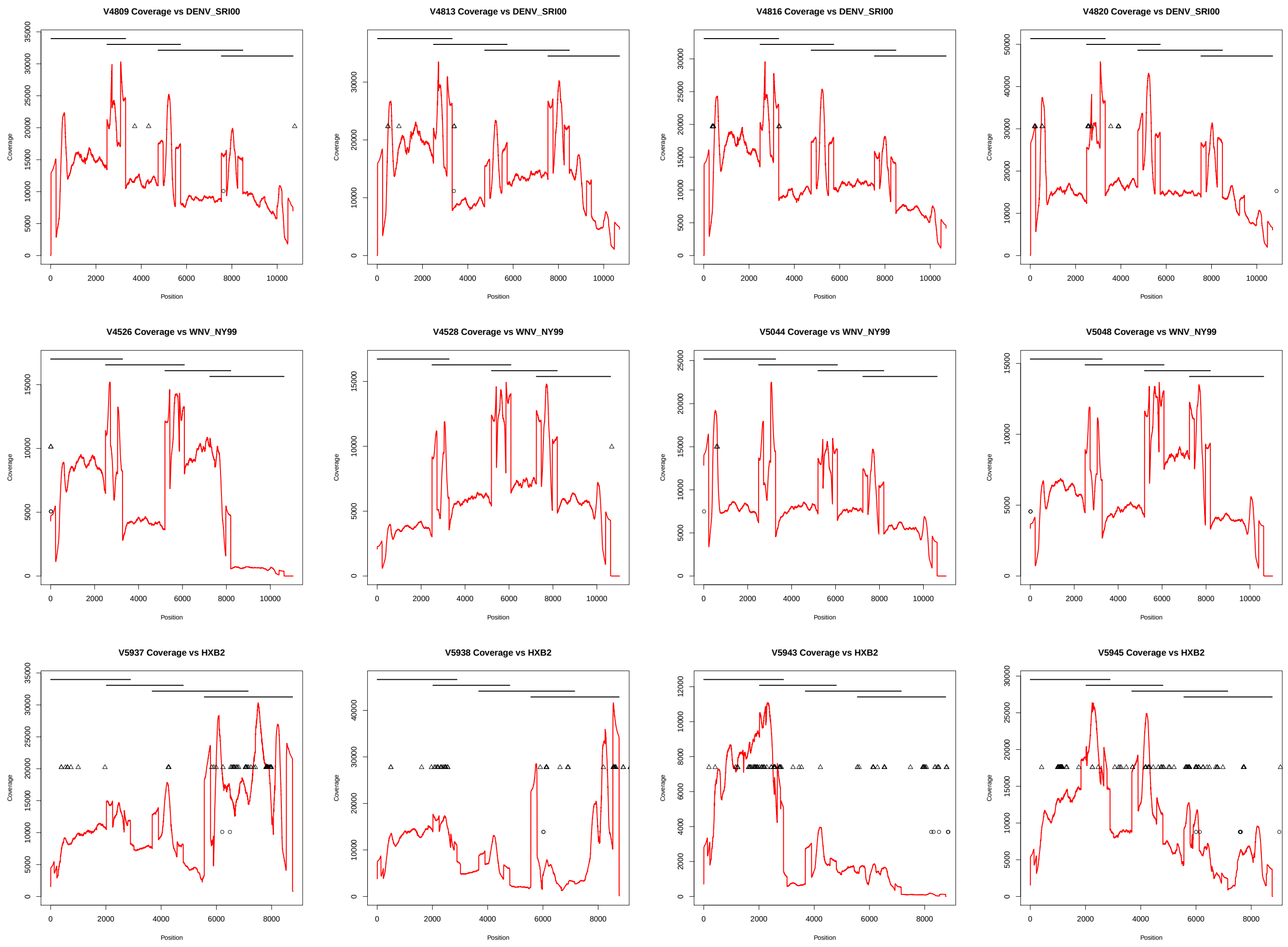


Supplementary Figure 1: Fold sequence coverage across the target region of Dengue, WNV, and HIV full length genomes. Alignments are to standard references (see Methods). Four horizontal lines on each figure represent amplicons used for generating the corresponding data set. Triangles and circles denote the non-dominant variant calls by *AV454* and *VICUNA*, respectively, with respect to the reference genomic position.

Supplementary Table 1: Datasets from Clinical WNV, DENV, and HIV Samples.

| Virus | V# | NCBI SRA & *VICUNA* assembly accession | Number of reads | % Reads aligning to standard reference | % Target region covered | Average coverage | (%) Divergence between reference and sample* |
|---|---|---|---|---|---|---|---|
| WNV | V4526 | XXXX | 305,162 | 95.28 | 100.00 | 6262.0 | 0.214 |
| | V4528 | XXXX | 322,134 | 95.01 | 100.00 | 6527.9 | 0.165 |
| | V5044 | XXXX | 434,800 | 95.11 | 100.00 | 8744.9 | 0.116 |
| | V5048 | XXXX | 316,880 | 95.04 | 100.00 | 6429.7 | 0.252 |
| DENV | V4809 | XXXX | 645,516 | 93.93 | 100.00 | 12975.8 | 5.770 |
| | V4813 | XXXX | 768,698 | 93.29 | 100.00 | 15456.8 | 5.751 |
| | V4816 | XXXX | 641,024 | 93.77 | 100.00 | 12951.8 | 5.760 |
| | V4820 | XXXX | 952,954 | 91.78 | 100.00 | 18733.4 | 5.829 |
| HIV | V5937 | XXXX | 568,380 | 87.77 | 100.00 | 12451.0 | 6.978 |
| | V5938 | XXXX | 453,648 | 90.52 | 100.00 | 9990.7 | 6.062 |
| | V5943 | XXXX | 134,894 | 92.60 | 100.00 | 3210.9 | 6.410 |
| | V5945 | XXXX | 440,260 | 89.98 | 100.00 | 10115.1 | 6.283 |

*This is calculated in the same way as non-dominant call rate.

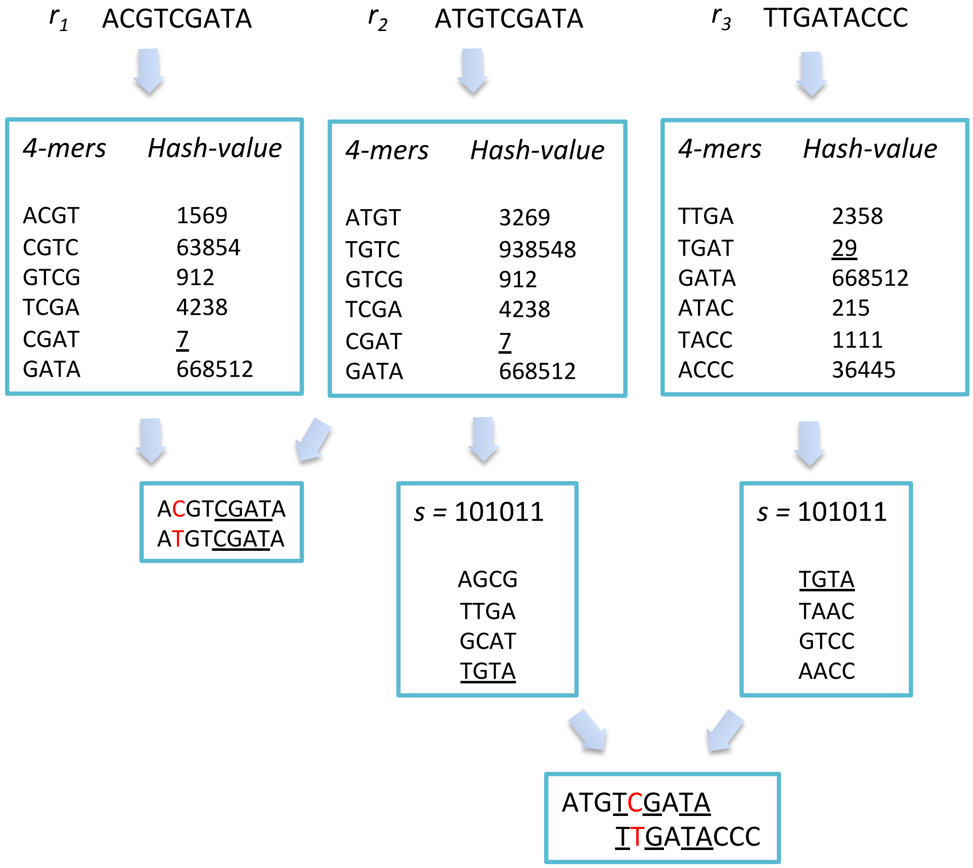


Supplementary Figure 2: An example of contig construction. The $k$-spectrum ($k = 4$) of reads $r_1$, $r_2$, and $r_3$ are computed and hashed to an integral space. $r_1$ and $r_2$ share a common min hash value of 7, and hence can be clustered and aligned. When we further use a gapped seed, 101011, where a '0' denotes an ignored position, to generate gapped-4-mers of $r_2$ and $r_3$, a common 4-mer "TGTA" can be identified, leading to the clustering of $r_2$ and $r_3$. In both cases, the 4-mers that lead to the clustering are underlined. We can see both techniques tolerate base differences (colored red) that may due to sequencing error or true variation. Note that the illustration of min hash technique in this example is a simplified version compared to the one used in our algorithm.

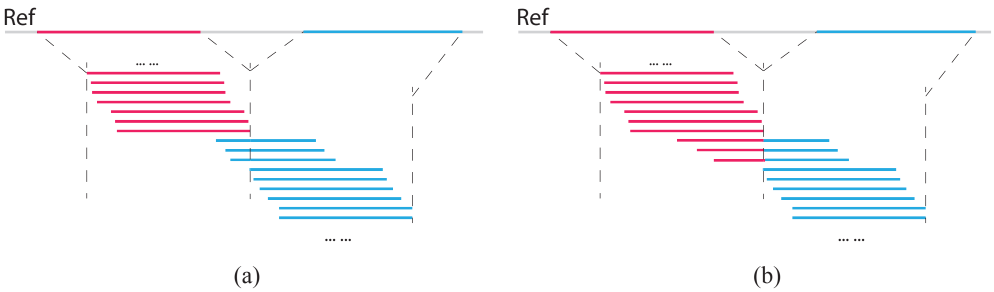


Supplementary Figure 3: Chimeric contig. Each read is represented as a short line, assigned with the same color as the fragment of the reference genome from which the read is sampled. Cause of chimeric contig due to (a) local homology among reads, or (b) chimeric reads, colored both red and blue, consisting of fragments from disjoint locations on the reference.

Supplementary Table 2: Large deletions in Clinical Samples.

| V# | Virus | RefStart | RefEnd | RegionStart | RegionEnd | Observed in 454 data? |
|---|---|---|---|---|---|---|
| V4809 | DENV | 3655 | 5341 | NS2A | NS3 | no |
| V4820 | DENV | 5270 | 8233 | NS3 | NS5 | no |
| | | 390 | 2713 | Caps | NS1 | no |
| | | 6236 | 7817 | NS3 | NS5 | no |
| | | 651 | 3000 | Memb | NS1 | no |
| V5937 | HIV | 6085 | 7420 | Env | Env | yes |
| | | 6309 | 6848 | Env | Env | yes |
| | | 6302 | 7348 | Env | Env | yes |
| V5938 | HIV | 5768 | 7546 | Env | Env | yes |
| | | 6441 | 7977 | Env | Env | yes |
| | | 5863 | 8247 | Env | Env | yes |
| | | 2468 | 4084 | Pol | Pol | no |
| V5943 | HIV | 2510 | 4402 | Pol | Vif | yes |
| V5945 | HIV | 6060 | 7498 | Env | Env | yes |
| | | 6362 | 7158 | Env | Env | yes |
| | | 4496 | 6316 | Vif | Env | yes |
| | | 2627 | 4546 | Pol | Vif | yes |

Supplementary Table 3: *VICUNA* Assembly Results for 454 Clinical Samples.

| Virus | V# | # Input reads | # Output contigs (≥ 350bp) | % Target region covered | # Contigs used for reference guided merging | % Target region covered by the longest contig | % Reads aligning to consensus | Non-dominant call rate (%) |
|---|---|---|---|---|---|---|---|---|
| WNV | V4954 | 40,942 | 9 | 100 | 1 | 100 | 92.55 | 0 |
| DENV | V4639 | 18,254 | 2 | 100 | 1 | 100 | 95.00 | 0 |
| HIV | V4139 | 28,963 | 1 | 100 | 1 | 100 | 98.53 | 0 |

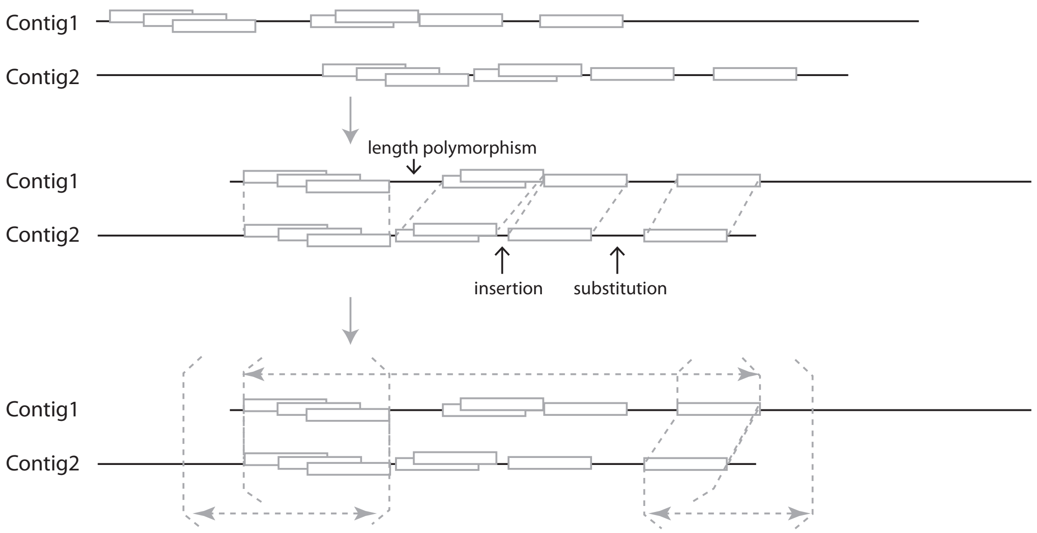


Supplementary Figure 4: Alignment of two contigs 1 and 2 that are represented as long lines. Common *k*mers between them are denoted as rectangles (top panel), these *k*mers are extended to form maximal common substrings (middle panel), which are forced to be aligned to each other. Inter-alignment regions may be resulted from length polymorphisms and sequencing errors (insertion, deletion and substitutions), which are further aligned using Needleman-Wunsch algorithm (bottom panel).

**Supplementary Algorithm 1** Contig construction via min hash and spaced-seed.

**Require:** $R = \{r_1, r_2, \ldots, r_n\}$, spaced-seed $s$

For each read $r_i \in R$, generate two min hash values, respectively, for its forward and reverse complementary strands.<br>
Cluster reads that share common min hash values to form initial contigs<br>
$\mathbf{D} \leftarrow \emptyset$<br>
**for** each $r_i \in R$ **do**<br>
    $\mathcal{S}_i \leftarrow \emptyset$<br>
    **for** $j = 0 \rightarrow |r_i| - |s| + 1$ **do**<br>
        $x \leftarrow$ apply $s$ to $r_i[j, j + |s| - 1]$<br>
        $\mathcal{S}_i = \mathcal{S}_i \cup \{\langle x, (i, j)\rangle\}$<br>
    **end for**<br>
    **if** $\exists S \in \mathcal{S}_i$ such that $S.key = S'.key$, where $S' \in \mathbf{D}$ **then**<br>
        Let $r'$ denote the read, where $S'.key$ belongs<br>
        Identify the two contigs $C_i$ (may not exist) and $C'$, where $r_i \in C_i$ and $r' \in C'$<br>
        **if** neither $C_i$ nor $C'$ contains both $r_i$ and $r'$ **then**<br>
            Add $r_i$ to $C'$<br>
        **end if**<br>
    **else**<br>
        $\mathbf{D} = \mathbf{D} \cup \{\mathcal{S}_i\}$<br>
        Create a singleton contig that contains only $r_i$<br>
    **end if**<br>
**end for**

**Supplementary Algorithm 2** Contig clustering via common reads.

Generate a 2-tuple $\langle id(r), id(C)\rangle$ for each read $r$ that is contained by contig $C$ and at least by one other contig. The results are stored in $\mathbf{M_{rc}}$<br>
**while** $\mathbf{M_{rc}}$ is not empty **do**<br>
    Sort contigs by the number of reads they contain in a decreasing order<br>
    Flag all contigs as unprocessed<br>
    **while** $\exists$ some unprocessed contig **do**<br>
        Identify the first one in the list, let it be $C$<br>
        Identify neighbors of $C$ using $\mathbf{M_{rc}}$.<br>
        Merge $C$ with its neighbors and flag all contigs involved as processed<br>
        Update $\mathbf{M_{rc}}$ by reassigning reads to new contigs when applicable<br>
    **end while**<br>
**end while**

**Supplementary Algorithm 3** Contig validation.

**Require:** an input contig $C$, parameters $max_d$, $max_{rt}$, $min_{ol}$

Initialize contig list $\mathbf{C}^{\dagger}$ to be empty
**repeat**
  $C_{cur} \leftarrow C$
  Generate consensus for $C_{cur}$
  Initialize contig $C_{rem} \leftarrow \emptyset$
  **repeat**
    **for** each read $r$ in $C$ **do**
      Measure the distance $d$ between $r$ and $\mathcal{C}$
      **if** $d > max_d$ **then**
        $C_{cur} = C_{cur} \setminus \{r\}$ and update consensus
        $C_{rem} \leftarrow C_{rem} \cup \{r\}$
      **end if**
    **end for**
  **until** no change was applied to $C$
  Add $C_{cur}$ to $\mathbf{C}$
  $C_{cur} \leftarrow C_{rem}$
**until** $C_{cur} = \emptyset$
**for** each contig $C \in \mathbf{C}$ **do**
  Generate the layout $(r_1, r_2, \ldots, r_{|C|})$ of $C$
  Calculate $\frac{n_b}{n_a}$ for each read in the layout
  Split $C$ at $r_i$ when either the overlap between $r_i$ and $r_{i-1}$ is $< min_{ol}$ or $\frac{n_b}{n_a} > max_{rt}$ for $r_{i-1}$
  Replace $C$ with the resulting contigs if split occurred
**end for**

† $\mathbf{C}$ stores the resulting list of contigs.

**Supplementary Algorithm 4** Contig extension.

**Require:** an input vector of contigs $\mathbf{C}$.

Sort $\mathbf{C}$ in an order of decreasing length
Generate a 2-tuple $\langle id(r), id(C) \rangle$ for each read $r$ contained in contig $C$. The results are stored in $\mathbf{M_{rc}}$
**while** existing more contigs to be processed **do**
  Select target contig $C_l \leftarrow$ the first element of $\mathbf{C}$
  Get neighbors $\mathbf{N}$ of $C_l$ via $\mathbf{M_{rc}}$
  Sort $\mathbf{N}$ in an increasing order of the number of paired-reads shared with $C_l$
  Compute delegates for $C_l$ and each contig in $\mathbf{N}$
  **while** $\mathbf{N}$ is not empty **do**
    $C_r \leftarrow$ the last element of $\mathbf{N}$
    Compare delegate $dg_l$ of $C_l$ with $dg_r$ (algorithm 5)
    **if** a significant prefix-suffix alignment is identified **then**
      Merge contig $C_l$ to $C_r$ & update $dg_r$
      Update $\mathbf{N}$ to include neighbors of $C_r$ & calculate delegates for newly included contigs
      Update $\mathbf{M_{rc}}$
    **end if**
    Remove the last element from $\mathbf{N}$
  **end while**
**end while**

**Supplementary Algorithm 5** Alignment of two sequences $s_0$ and $s_1$.

**Require:** parameters $k$, $min_{ol}$, $min_s$, $max_d$, $max_{oh}$

Identify every common $k$mer $x$ between $s_0$ and $s_1$, and record $x$ along with its start positions $(p_s^0, p_s^1)$ as a 2-tuple $\langle x, (p_s^0, p_s^1)\rangle$ in array $\mathbf{A}$
Sort $\mathbf{A}$ in an increasing order with respect to $p_s^0$
Flag each entry of $\mathbf{A}$ as unprocessed.
**for** $i = 0 \rightarrow |\mathbf{A}| - 1$ **do**
  **if** $\mathbf{A}[i]$, the $i^{th}$ element of $\mathbf{A}$, is flagged as unprocessed **then**
    $a \leftarrow \mathbf{A}[i]$
    Add $\langle p_s^0, p_e^0, p_s^1, p_e^1\rangle$ to $\mathbf{V}^\dagger$, where $p_j^i$ is the start $(j = s)$ or end $(j = e)$ positions of $a.key$ on $s_i$ $(i = 0, 1)$
    **for** $j = i \rightarrow |\mathbf{A}| - 1$ **do**
      $b \leftarrow \mathbf{A}[j]$
      **if** $b.key$ starts within $a.key$ **then**
        Update $\mathbf{V}$ if the two $k$mers can be joined to be a common substring of $s_0$ and $s_1$
        Flag $b.key$ as processed
      **else if** $b.key$ starts after $a.key$ but within $max_d$ **then**
        Add the coordinates of $b.key$ to $\mathbf{V}$
        $a \leftarrow b$
      **else**
        Generate prefix-suffix alignment between $s_0$ and $s_1$ relying on $\mathbf{V}$
        If a valid alignment can be identified, accept this alignment and exit
      **end if**
    **end for**
  **end if**
**end for**

$^\dagger\mathbf{V}$, initially empty, is an array of 4-tuples: $\langle i_0, i_1, i_2, i_3\rangle$, where $s_0[i_0, i_1] = s_1[i_2, i_3]$
